# Psychiatry

**Published:** 1905-05-13

**Authors:** 


### PSYCHIATRY.

**Insanity as a Biological Problem.**—The great attention paid in recent years to biological problems and heredity has made itself felt even in the domain of mental pathology. The question of the hereditary transmission of an insane or of a neuropathic taint has been keenly discussed by alienists and neurologists. Dr. John MacPherson,[1] Commissioner in Lunacy for Scotland, draws attention to several questions of great practical importance in connection with insanity, heredity, and variation. Insanity comprises different diseases which have one characteristic in common—that they have as their result or accompaniment a more or less grave or dangerous disturbance of the normal processes of thought, feeling, and conduct. Taking various groups of insanity Dr. MacPherson applies the biological method to the study of their relation to human character, in so far as they are hereditarily transmitted. Those dealt with include confusional or toxic insanity, the recurrent insanities, dementia, pre-senile and senile forms of insanity. Confusional insanity comprises the various deliria of fevers, alcoholism, puerperal forms of insanity, dementia præcox, and general paralysis. Its typical form is the toxic delirium of fevers, known to all medical men and to the majority of the lay public. Briefly, delirium is characterised by illusions and hallucinations of the senses, by confusion and hurry of thought, by incoherent speech, by fragmentary delusions, and by physical restlessness. "Whatever the cause of the delirium, at whatever age it occurs, or whatever its form, there is always present a combination of the symptoms mentioned. . . . In some of the deliria secondary features, or different combinations of the same features are present . . . and by a familiarity with these features we can tell without much difficulty acute alcoholism from the delirium of, say, enteric fever, and that of the latter from small-pox." Delirium is thus a result of acute toxic infection accompanied by pyrexia or fever. Some persons scarcely manifest it however much the system may be poisoned, while others become delirious on apparently trivial occasions. It is interesting to note the extreme liability of children to delirium. "That fact, along with the fact that the liability varies so greatly among adults, teaches us that inherent variation exists among individuals." Mental confusion, as understood by alienists, is less acute and more prolonged in its symptoms than delirium proper; in fact, it might be described as "a chronic manifestation of delirium." There are illusions and hallucinations of the senses, confusion of thought, incoherency of speech, but no constant emotional state. Thus we find mental confusion succeeding influenza, typhoid fever, erysipelas, and phthisis. It also occurs in susceptible subjects as the result of exhaustion after severe shock, after serious accidents, and, finally, after prolonged mental or physical strain. But we must note the fact that it succeeds such causative factors in highly predisposed and neuropathic subjects only. Alcohol is one of the prolific causes of a large group of confusional insanities, as shown in delirium tremens, *mania à potu*, and alcoholic dementia with or without delusions of a distinctive type. The insanities connected with pregnancy and child-bearing are almost wholly confusional. The term

"puerperal mania" is a misnomer. Adolescent dementia, also called dementia præcox, "affects young persons of neurotic disposition almost without exception . . . according to Kraepelin heredity has been ascertained to be present in 70 per cent. of all the cases. Over 60 per cent. of the cases are under 25 years of age . . . Many of the subjects are intellectually bright, some are even brilliant; others, about 7 per cent, of the whole, are weak-minded. This is not to be wondered at, for the neurotic disposition is not limited to any intellectual grade." Dementia præcox, which is a slow progressive disease, is produced by two factors, viz., a predisposing cerebral weakness and a physical intoxication. The pathological effect of the toxin is so severe under the circumstances that only about 8 per cent. of the cases make a good recovery. Delirium, confusion, and stupor, adds Dr. MacPherson, constitute the three components of confusional insanity. Stupor follows the mental confusion of alcoholism, of post febrile states, of shock and exhaustion, but more especially of that very grave mental affection of puberty and adolescence which is known as dementia præcox. This group of insanities, the confusional-toxic group, "is a distinct group characterised by the mental symptoms mentioned, and caused by definite well-known factors, viz., poisoning of the nervous system, and exhaustion of various kinds." The occurrence of toxæmia in cases of this class have been proved in recent years by systematic examination of the blood of patients, a condition of hyperleucocytosis with special increase in the number of polymorphonuclear cells being the characteristic sign of toxæmia.

**Recurrent Insanity.**—A sharp clinical line separates the confusional group of insanities from the "recurrent insanities." The recurrent insanities are degenerative in origin; that is to say, they occur in persons in whom the presence of an inherited neuropathic constitution is well-marked. The hereditary tendency of the subjects is very high—higher than in any other group of mental affections that we know of. "Like the general neuroses (epilepsy, hysteria, etc.), their origin is obscure, and they occur without any apparent external cause." They manifest the inexplicable and uncontrollable periodicity of the functional neuroses. They occur in most cases for the first time in early adult life, and they have a characteristic tendency to recur. In marked contradiction to the toxic insanities emotional disturbance is the dominant mental symptom. This group of the insanities comprises the time-honoured forms mania, circular insanity, and melancholia, but, owing to the astonishingly simple fact that these are not separate entities, as pointed out by Kraepelin, but different phases in the periodic march of one syndrome which Kraepelin aptly names manic-depressive insanity, they may be separately considered. Examinations of the blood show an essential clinical difference between the confusional-toxic, and the recurrent manic-depressive groups of insanities. "There is a toxin in the blood in the recurrent manic-depressive group of insanities which rises and falls with the occurrence of the attacks; which seems to disappear, exactly as the malarial toxin does, in the intervals between the attacks; and which exercises a less deleterious influence upon nervous tissue than the toxins of the confusional insanities do." In the meantime all that we can say, concludes Dr. MacPherson, is that neither mania, melancholia, nor paranoia ever results from external infections, intoxications, or visceral diseases. Dr. Dercum says: "To speak of delirium as a mania because it happens to be attended by excitement is certainly a gross misuse of terms; . . . to designate a confusional insanity as a melancholia merely because the delusions are distressing or painful is equally unscientific." Paranoia is, says MacPherson, a continuous, not a periodic insanity. It is, however, allied to manic-depressive insanity in that it is a degenerative, highly neuropathic condition. There are many varieties of paranoia, many of them by no means typical, but they are all reducible to the following typical form:—The disease begins with a phase of depression (lowering of the organic feeling of well-being) extending over many years, and this is accompanied and followed by a system of delusions, slowly and logically evolved. The delusions are persecutory, and at first distress the patient. Long and continued evolution of delusions results in alteration of personality, the delusions now become of a different character—that is, grandiose. This evolutionary history characterises paranoia. Manic-depressive insanity and paranoia are in many respects, says MacPherson, allied to the neuroses. "They depend upon no external causes for their origin any more than epilepsy and hysteria do." The insanities of morbid impulse and obsession are more distantly related to the class of recurrent insanities. Of the hereditary and neuropathic origin of this group there can be little doubt. It is pretty generally assumed that they are manifestations of a neurasthenia or rather a prenasthenia, but this does not solve the problem of their origin. The imperative ideas which form the basis of obsessions are as varied as there are thoughts in the human mind. The common obsessions are those of the nature of fear (*e.g.* agoraphobia) and indecision (*folie du doute*). Some lead to anti-social and dangerous acts, in which case the question of responsibility and of asylum care may have to be considered. The insanities of old age commence to develop earlier in life than is generally supposed. Thus we find in late middle life a "climacteric melancholia" begin to appear. It is a prolonged, very emotional, and somewhat unfavourable form. This is the true melancholia. "There is no melancholia of early life, and no mania of late life." With the exception of involutional melancholia of the climacteric and senile periods, mania and melancholia as isolated and uncomplicated entities only occur as phases of the great mania-melancholia syndrome which, as a rule, makes its appearance for the first time in early adult life. At a later age than the climacteric the forms of insanity become of the senile type; in other words, they are dementias of involution. The symptoms are again confusional, complicated with loss of memory, with emotional disturbance, with illusions and hallucinations of the senses, or with vague systematised delusions.

[1] Jour. of Mental Sci., April 1905.

(*To be continued.*)

